# The Loss of *SMAD4/DPC4* Expression Associated with a Strongly Activated Hedgehog Signaling Pathway Predicts Poor Prognosis in Resected Pancreatic Cancer

**DOI:** 10.7150/jca.30883

**Published:** 2019-07-10

**Authors:** Jin-Zhi Xu, Wen-Quan Wang, Wu-Hu Zhang, Hua-Xiang Xu, He-Li Gao, Shi-Rong Zhang, Chun-Tao Wu, Shuo Li, Hao Li, Jin Xu, Xian-Jun Yu, Liang Liu

**Affiliations:** 1 Department of Pancreatic Surgery, Fudan University Shanghai Cancer Center, Shanghai, China.; 2 Department of Oncology, Shanghai Medical College, Fudan University, Shanghai, China.; 3 Shanghai Pancreatic Cancer Institute, Shanghai, China.; 4 Pancreatic Cancer Institute, Fudan University, Shanghai, China.

**Keywords:** pancreatic ductal adenocarcinoma, Hedgehog signaling pathway, driver genes, *SMAD4/DPC4*, prognosis

## Abstract

**Background**: Pancreatic ductal adenocarcinoma (PDAC) progression is mediated by mutations in driver genes and a complex stroma that is mainly dependent on the Sonic hedgehog (Shh) signaling pathway. However, the association between driver genes and Shh-pathway proteins and their potential prognostic significance remain unclear.

**Methods**: We analyzed protein expressions of the *KRAS, TP53, SMAD4*, and *CDKN2A/P16* driver genes and the Shh-pathway molecules, including Shh, glioma-associated oncogene (Gli) 1, Gli2, and smoothened (SMO) by immunohistochemistry using tissue microarrays in 237 patients with resectable PDAC and statistically determined their prognostic significance.

**Results**: *SMAD4*^lost^ mutation was associated with shorter survival outcomes [overall survival (OS): Hazard ratio (HR) 1.887, *p* < 0.001]; recurrence-free survival (RFS): HR 1.886, *p* < 0.001) and abnormal p53 immunolabeling was associated with poor OS (HR 1.436, *p* = 0.011) in patients with PDAC. The mutational status of p16 had no effect on patient survival. High levels of SMO and Gli1 expression were associated with poor survival outcomes in both univariate and multivariate analyses. Pearson's χ^2^ test showed a medium correlation between the *SMAD4*^lost^ mutation and Shh (R = 0.343) and Gli1 (R = 0.505) expression levels (*p* < 0.001). Patients with the *SMAD4*^lost^ mutation and high levels of Shh and Gli1 expression showed the poorest survival outcomes (RFS: HR 2.976; OS: HR 3.598; *p* < 0.001 for both) compared with other patients in the study.

**Conclusion**: Loss of *SMAD4* associated with a strongly activated Shh pathway resulted in poor survival outcomes in patients with resected PDAC.

## Introduction

Pancreatic cancer, currently ranked as the fourth leading cause of cancer-related deaths, is a lethal disease with an overall 5-year survival rate of about 8% 1. It is projected to become the second leading cause of cancer-related deaths by 2030 2. Surgical resection, the only potentially curative therapy for patients with pancreatic ductal adenocarcinoma (PDAC), offers a 5-year survival rate of 10-18% 3. The aggressive malignancy of this disease and the lack of effective treatment have led to a dismal prognosis 4, 5. An effective biomarker to predict postoperative prognosis in PDAC is still lacking.

PDAC is characterized by a dense, heterogeneous stroma composed of fibroblasts, stellate cells, extracellular matrix, and immune cells and the Sonic hedgehog (Shh) signaling pathway is crucial for the formation of this dynamic compartment 6, 7. The Shh signaling pathway, first discovered in 1980^8^, has an early and critical role in the genesis of pancreatic cancer 6, 7. The Shh protein binds and inactivates the membrane receptor, patched 1 (PTCH1) that normally inhibits the 7-transmembrane protein, Smoothened (SMO). SMO, in turn, activates the glioma-associated oncogene (Gli) transcription factors, Gli1 and Gli2 9, and turns on the Shh signaling pathway, which promotes cancer cell proliferation. Despite the key role of the Shh pathway in pancreatic cancer, the prognostic and predictive values of currently available Shh molecular markers are not yet reliable and require further evaluation with large patient populations 10.

It is increasingly evident that tumors do not exist in isolation and the tumor-environment crosstalk plays an important role in cancer progression 10. PDAC is characterized by several genetic alterations in key genes, such as mutations that activate the *KRAS* oncogene and inactivate the tumor suppressor genes, *TP53,* mothers against decapentaplegic homolog 4 (*SMAD4/DPC4*)*,* and cyclin dependent kinase inhibitor 2A (*CDKN2A/P16*) 7, 11-14. We have previously shown that pancreatic tumor cells closely interact with their immune microenvironment 15. However, it is not yet clear whether specific driver gene mutations are involved in stroma formation. Some studies have shown potential effects of driver genes on the Shh signaling pathway. The *KRAS* oncogene activates Shh signaling in PDAC cells 16 and loss of *TP53* may activate Shh signaling in many cancers 17. TGFß-Smads signaling positively regulates *GLI1* transcription 18. These results highlight the importance of the need for a detailed understanding of tumor-stroma interactions.

The crosstalk between tumor and tumor microenvironment plays an important role in tumor progression and is considered to be involved in treatment and prognosis. In this study, we investigated potential associations between major driver gene mutations and the Shh signaling molecules that promote the formation of a fibroblast-rich stroma in PDAC. We also evaluated their association with clinicopathological features and prognosis. The status of major driver genes and Shh signaling proteins may be used as molecular prognostic markers to make better therapeutic decisions and may provide insights into tumor-stroma interactions.

## Methods

### 1. Selection of patients

We collected data from 237 patients diagnosed with PDAC who underwent curative-intent surgery and adjuvant chemotherapy at the Pancreatic Cancer Institute of Fudan University from January 2010 to December 2013. All patients in the study were diagnosed to have resectable PDAC, namely there is no arterial tumor contact [celiac axis (CA), superior mesenteric artery (SMA), common hepatic artery] and there is no tumor contact with the superior mesenteric vein (SMV) or portal vein (PV) or ≤ 180-degrees contact without vein contour irregularity 5. Exclusion criteria included preoperative chemotherapy and/or radiotherapy, macroscopically incomplete resection (R2), pancreatic tumor histology other than ductal adenocarcinoma. Patients who died of postoperative complications within 30 days after surgery were also excluded. Overall survival (OS) was calculated as the interval between the date of surgery and the date of death or the last follow-up visit. Recurrence-free survival (RFS) was defined as the interval between the date of surgery and the date of tumor recurrence or the last follow-up visit. All patients were monitored until December 2017. A TNM stage was assigned to each patient according to the 8th edition of the Union for international cancer control (UICC) staging system for pancreatic cancer 5. We used the recommended upper limit of 37 U/mL for CA19-9, the diagnostic biomarker for pancreatic cancer 19. This study was approved by the appropriate research Ethics Committees, and informed consent was obtained from all patients.

### 2. Immunohistochemistry and tissue microarrays (TMAs)

Immunostaining was performed using TMAs (Shanghai Biochip Company, China), which were constructed as described previously 15 using two tissue cores (1.5-mm diameter) taken from representative areas of each formalin-fixed, paraffin-embedded tumor specimen. Previous studies have shown that the immunohistochemical labeling of Kras, p16, p53, and Smad4 reflects their respective genetic status in PDAC 20-22. The antibodies and the concentrations used for immunostaining are provided in Table S7.

### 3. Measurement of marker positivity in cell populations

TMA slide images were captured as high-resolution digital files. Immunostainings were independently evaluated by two pancreatic pathologists who were blinded to the clinical data and any discrepancy in their analysis was resolved by consensus. Islet cells were used as an internal control for immunolabeling. Immunohistochemical labeling of Smad4 and p16 were scored as intact (positive), which indicated the presence of an intact gene, or lost (negative), which indicated a loss of function mutation or deletion of the gene 22, 23. p53 expression was considered abnormal in two scenarios: 1) a virtual absence (<5%) of p53 immunolabeling in neoplastic cells compared with adjacent normal tissue, which suggested the presence of an intragenic deletion, nonsense or frameshift mutation; and 2) robust nuclear accumulation of immunolabeled protein in ≥30% of neoplastic cells compared with adjacent normal cells 11, 24. Immunohistochemistry of the driver genes are shown in Fig. S1.

The expression levels of Shh and the downstream factors, Gli1, Gli2, and SMO, were defined as follows: The percentage positivity was scored as 0 (<5%), 1 (5%- 25%), 2 (25%-50%), 3 (50%-75%), or 4 (>75%). The staining intensity was score as 0 (no staining), 1 (weakly stained), 2 (moderately stained), or 3 (strongly stained). Expression levels were determined using the following formula: immunohistochemistry (IHC) score = percentage score × intensity score. An IHC score > 6 was defined as high level of protein expression 25.

### 4. Statistical analysis

IBM SPSS Statistics software version 23 (IBM Corporation, USA) was used to organize and analyze data. Continuous variables were expressed as median and range, and categorical variables were compared using the χ^2^ test or Fisher's exact test. OS and RFS were estimated using the Kaplan-Meier method and compared using the Cox model. The Pearson χ^2^ test or the Fisher exact test was used to correlate Smad4, Gli1, and SMO expression with clinicopathologic features. The concordance index (C-index) and Akaike information criterion (AIC) were used to compare the accuracies of predictive models. Results were considered statistically significant for *p* < 0.05.

## Results

### 1. Clinicopathological characteristics

**Fig 1:**
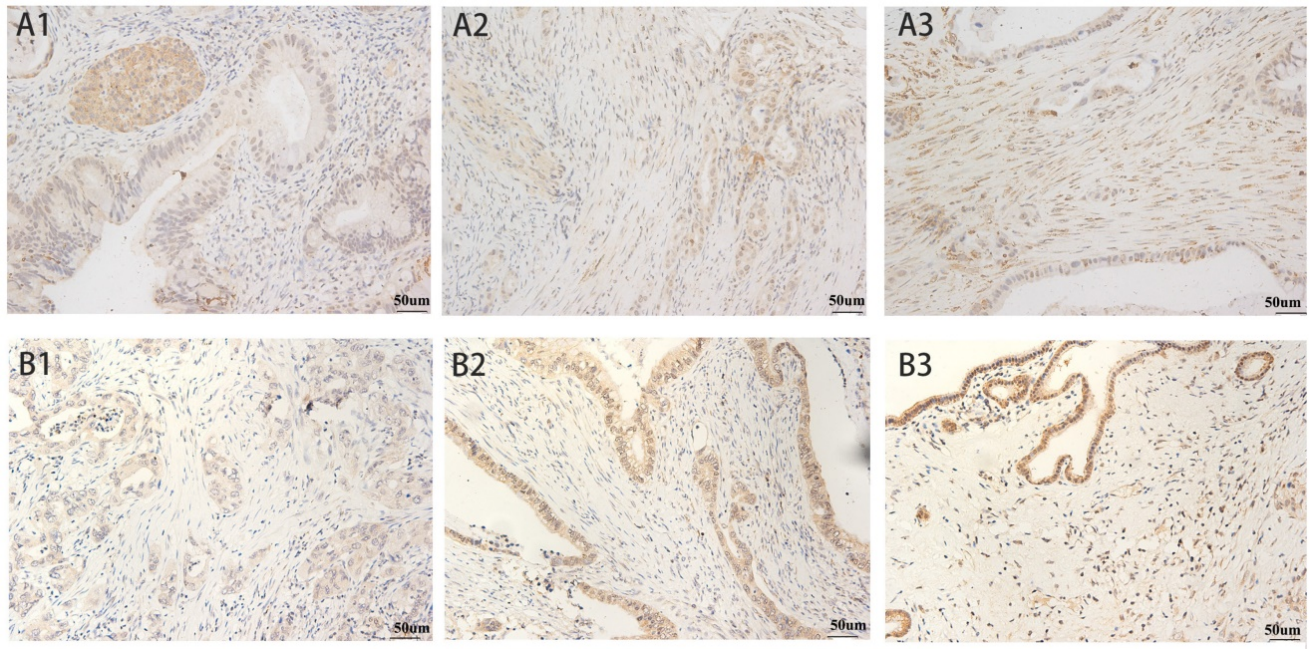
SMO and Gli1 Expression in Pancreatic Cancer. SMO: **A1)** weak expression, **A2)** moderate expression, and **A3)** intense expression in tumor cells and stroma. Gli1: **B1)** weak expression, **B2)** moderate expression, and **B3)** intense expression in stroma. All magnification = 400×. Positive staining appears brown.

The patient and tumor characteristics of 237 patients with resectable PDAC in this study are listed in Table S1. Immunohistochemical staining of common driver genes and the Shh-pathway proteins are shown in Table S2. Lack of Smad4 and p16 immunolabeling were observed in 168 (70.9%) and 177 (74.7%) patients, respectively. Abnormal immunolabeling of p53 was detected in 147 (62.0%) patients. All patients scored positive for immunostaining of Kras. Immunolabeling of Shh-pathway molecules showed that SMO was localized in the cytoplasm and the cell membrane of pancreatic tumor cells and Gli1 was localized in the cytoplasm and the nucleus. High levels of SMO and Gli1 expression was found in 62.0% and 48.1% of patients, respectively (Fig. 1). Shh was mainly localized in the cytoplasm of cancer cells and 43% of the patients (102 of 237) showed a high level of Shh expression. Gli2 was localized in the cytoplasm and nucleus of cancer cells and 57% of patients showed a high level of Gli1 expression (Fig. S2).

### 2. Survival analysis

Of the 237 patients with PDAC at the start of the study, only 9 were alive at the census date (December 2017). The median OS was 12.1 months, with 1-, 3-, and 5-year survival rates of 51.9%, 16.9%, and 8.8%, respectively. During the study period, recurrent disease occurred in all 237 patients. We performed a survival analysis to study the status of driver genes and Shh-pathway molecules and the clinicopathological characteristics in relation to OS or RFS in patients with PDAC (Table 1). Univariate Cox analysis showed that loss of Smad4 in patients resulted in worse OS [Hazard ratio (HR) 1.887, *p* < 0.001] and RFS (HR 1.886, *p* < 0.001) compared with patients with intact Smad4. Patients with abnormal p53 immunolabeling showed poor OS (HR 1.436, *p* = 0.011). The genetic status of p16 showed no effect in survival outcome (OS *p* = 0.739 and RFS *p* = 0.599) in patients with PDAC. Analysis of key molecules of the Shh pathway indicated that patients with high levels of Gli1 and SMO expression showed poor OS (Gli1: HR 1.988, *p <* 0.001; SMO: HR 1.411, *p =* 0.013) and RFS (Gli1: HR 1.716, *p <* 0.001; SMO: HR 1.436, *p =* 0.013). We found no association between the expression levels of Shh and Gli2 and patients' survival outcome.

**Fig 2:**
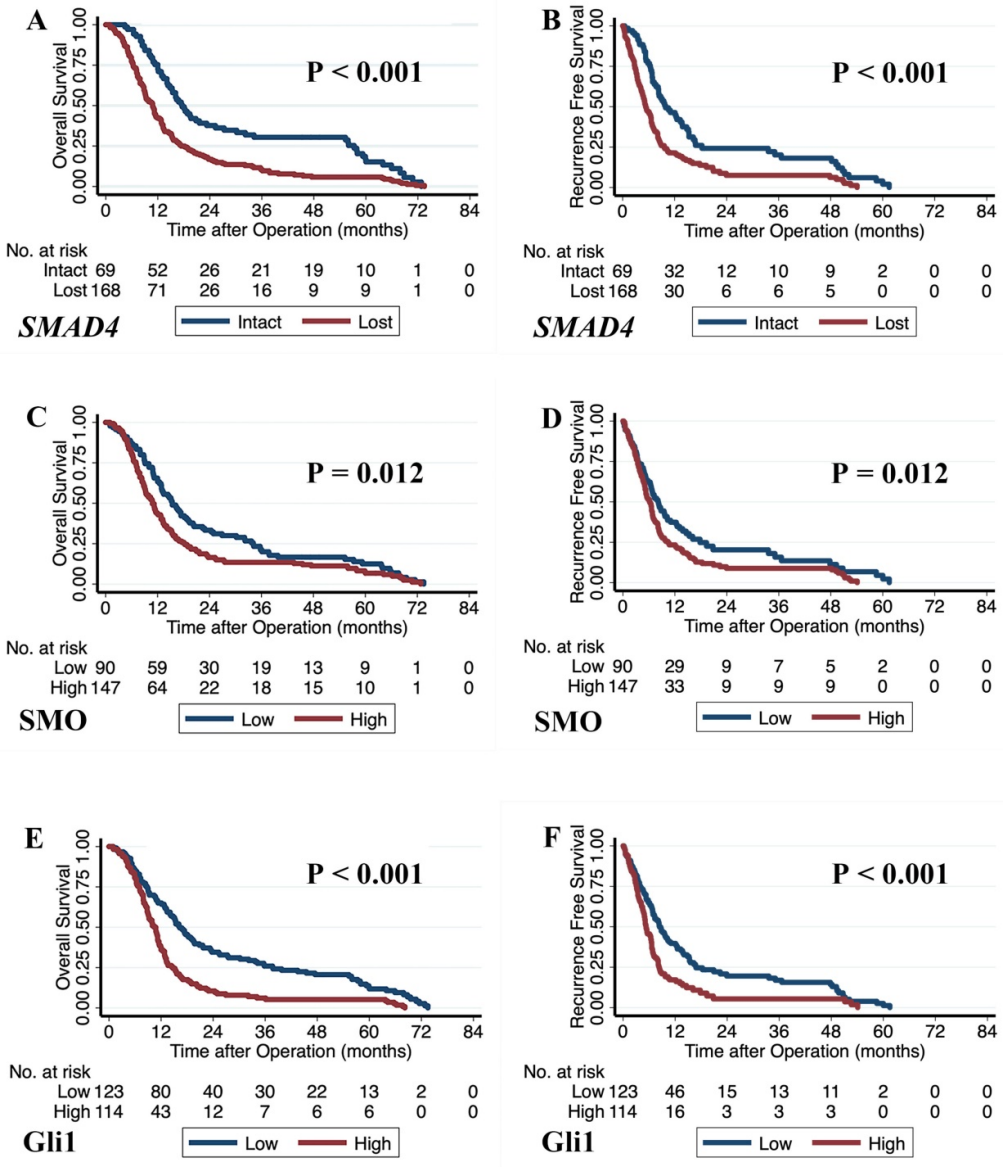
The Kaplan-Meier curves based on the genetic status of *SMAD4* and the expression levels of SMO and Gli1. **A)** Overall survival curves based on the genetic status of *SMAD4*. **B)** Recurrence-free survival curves based on the genetic status of *SMAD4*. **C)** Overall survival curves based on SMO protein expression. **D)** Recurrence-free survival curves based on SMO protein expression. **E)** Overall survival curves based on Gli1 protein expression. **F)** Recurrence-free survival curves based on Gli1 protein expression. '*p*' values were calculated by log-rank test.

We constructed multivariate models using Cox proportional hazards analysis with significant factors (*p* < 0.05) obtained from the univariate analysis (Table 1). Our results showed that loss of Smad4 immunolabeling was an independent prognostic factor for shorter OS (HR 1.551, *p* = 0.045) and a borderline significant prognostic factor for shorter RFS (HR 2.045, *p* = 0.074). Poor survival outcomes for patients were also associated with high levels of Gli1 (OS: HR 1.541, *p* = 0.020 and RFS: HR 1.590, *p* = 0.002) and SMO expression (OS: HR 1.782, *p* = 0.001; RFS: HR 1.776, *p* = 0.001). The Kaplan-Meier curves with log-rank test for the prognostic factors, including Smad4, SMO, and Gli1, are shown in Fig. 2.

We correlated the clinicopathological characteristics with patient survival outcomes, and poor overall survival outcomes were seen in male patients (HR 2.208, p < 0.001), patients with high CA19-9 (HR 3.190, *p* < 0.001), patients with tumors in the body and tail of pancreas (HR 1.821, *p* < 0.001), and patients with lymph node metastasis (HR 1.638, *p* = 0.001) and venous invasion (HR 2.020, *p* < 0.001) of tumors (Table 1).

### 3. Association of Smad4, Gli1 and SMO expression with the clinicopathologic features

Altered Smad4, Gli1, and SMO protein expression that were associated with significantly poor patient outcomes were correlated with the clinicopathological characteristics seen in patients with PDAC using the χ^2^ test (Table S3). We found that loss of Smad4 immunolabeling was associated with female patients (*p* = 0.015), patients with serum levels of CA19-9 > 37 U/mL (*p =* 0.003), patients with tumors in the body and tail of pancreas (*p* < 0.001), patients with a tumor size >2 cm (*p* = 0.028), and patients grouped into the T category of UICC classification (*p* = 0.040). High levels of Gli1 expression was significantly associated with patients with tumors in the body and tail of pancreas (*p =* 0.006), patients with poorly differentiated tumor tissues (*p* = 0.001), and patients grouped into the T3-4 category of UICC classification (*p =* 0.026). High SMO protein levels were significantly associated with female patients (*p* = 0.001), patients grouped into the N1-2 category of UICC classification (*p =* 0.001), and patients in Stage II-III of the American joint committee for cancer (AJCC) classification (*p =* 0.003).

### 4. Association between Shh-pathway molecules and driver genes

To test the hypothesis that specific driver gene mutations may influence the tumor microenvironment, we analyzed the relationship between the driver genes and Shh signaling molecules that we had found to be strongly associated with patient outcomes. Our results showed a moderate correlation between the loss of Smad4 and high expression levels of Shh (*p <* 0.001, Pearson's R = 0.343) and Gli1 (*p <* 0.001, Pearson's R = 0.505) (Table S4). We found no other significant correlation between the driver genes and the Shh-pathway molecules (Table S8).

### 5. Predictive model based on Shh pathway activation and driver gene mutations

**Fig 3:**
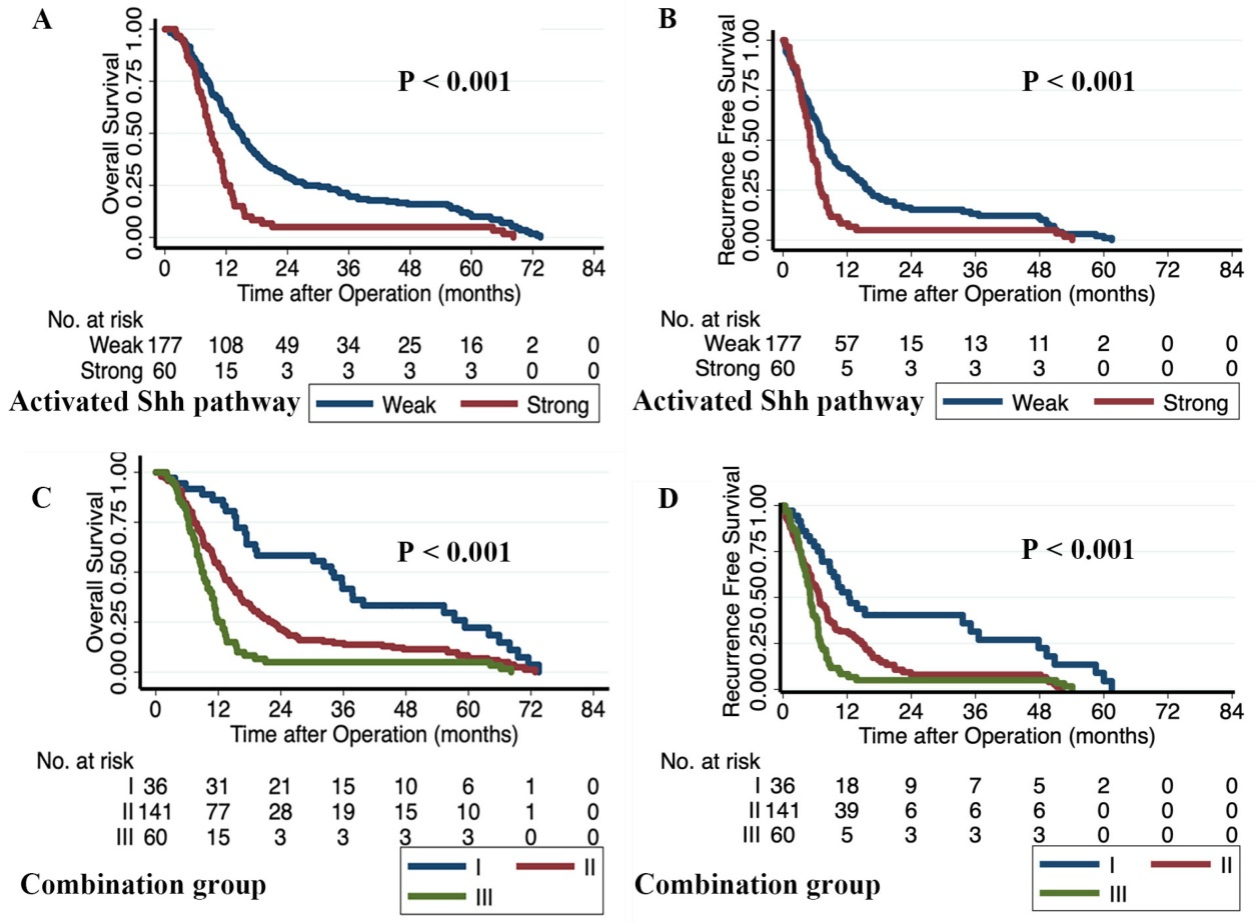
The Kaplan-Meier survival curves of the activated-Shh-pathway group and the integrated model group. Patients with Gli1^high^ and SMO^high^ were classified in the activated-Shh-pathway^strong^ group; all others were assigned to the activated-Shh-pathway^weak^ group. **A)** Overall survival curves based on the level of activation of the Shh pathway. **B)** Recurrence-free survival curves based on the level of activation of the Shh pathway. The integrated model group consists of three subgroups: group I, activated-Shh-pathway^weak^/*SMAD4*^intact^; group II, activated pathway^weak^/*SMAD4*^lost^ or activated-Shh-pathway^strong^/*SMAD4*^intact^; group III, with activated-Shh-pathway^strong^ /*SMAD4*^lost^.** C)** Overall survival curves based on the integrated model subgroups. **D)** Recurrence-free survival curves based on the integrated model subgroups. '*p*' values were calculated by log-rank test.

We constructed a predictive model to evaluate the combined prognostic capabilities of Smad4, Gli1, and SMO, which were all independent prognostic factors for OS in patients with PDAC. We classified patients with Gli1^high^ and SMO^high^ in the activated- Shh-pathway^strong^ group. All other patients were assigned to the activated-Shh-pathway^weak^ group. All patients were further divided into three integrated model subgroups: group I contained 36 patients with activated-Shh-pathway^weak^/Smad4^intact^; group II contained 141 patients with activated-Shh-pathway^weak^/*SMAD4*^lost^ or activated-Shh-pathway^strong^/*SMAD4*^intact^; and group III contained 60 patients with activated-Shh-pathway^strong^/*SMAD4*^lost^. The three subgroups showed significantly different OS and RFS and Group III showed the poorest survival outcome (RFS: HR 2.976, *p* < 0.001; OS: HR 3.598, *p* < 0.001) compared with the other two groups (Table 2). The Kaplan-Meier survival curves for the activated-Shh-pathway groups and the integrated model subgroups are shown in Fig. 3. Multivariate analysis showed that activated-Shh-pathway^strong^ /*SMAD4*^lost^ (group III) was an independent prognostic factor for poor RFS (HR 2.853, *p* < 0.001) and OS (HR 3.309, *p* < 0.001) (Table S5). Comparison of prognostic strengths revealed that the prognostic value of the integrated model was stronger than that of the individual variables. The integrated model group showed a higher concordance index (C-index; OS 0.6220, RFS 0.6076) compared with Shh-Pathway activation level (OS 0.5684, RFS 0.5548) and *SMAD4* status (OS 0.5862, RFS 0.5830) and a lower AIC (Integrated model: OS 2033, RFS 1914; Shh-Pathway activation level: OS 2045, RFS 1922; and *SMAD4*: OS 2052, RFS 1927) (Table S6).

## Discussion

Our study showed that loss of *SMAD4/DPC4* and a strong activated Shh pathway due to high expression levels of Gli1 and SMO resulted in a poor prognosis for patients with PDAC. These potential prognostic factors also correlated with clinicopathological features that were known to result in poor survival outcomes for patients with PDAC. Moreover, our study revealed that the driver genes mutation of tumor cell might program the tumor microenvironment, and the expressions of Shh and Gli1 were found to be related to the status of* SMAD4/DPC4*. And the integrated model based on the combination of these potential prognostic factors had a stronger prognostic value. We showed a correlation between the genetic status of *SMAD4* and the expression levels of Sonic hedgehog pathway proteins in patients with pancreatic adenocarcinoma. This association may serve as a potential prognostic marker for pancreatic cancer.

Studies have shown that driver gene mutants and Shh-pathway molecules are linked to pancreatic cancer prognosis 10, 25. The proportion of patients in our study with mutated *SMAD4/DPC4* (70.9%)*, CDKN2A/p16* (74.7%)*,* and *TP53* (62%) was similar to previously reported values (54.7% of *SMAD4/DPC4* 25, 67.3% of *CDKN2A/p16*, and 50-70% of *TP53* 26). The prognostic implications of p53 mutations remain unclear 23, 26-28. Although our univariate regression analysis showed that abnormal labeling of p53 was significant for OS, we were unable to confirm it by multivariate analysis. Previous studies 24, 29 have shown conflicting results on the correlation of *CDKN2A/p16* mutational status on patient survival. Our results show no such correlation, which may be due to an almost universal inactivation of the *CDKN2A* gene in pancreatic cancer partly because of methylation 7. Consistent with previous reports 15, 24, 28-30, we found that loss of *SMAD4/DPC4* was significantly associated with shorter OS and RFS in patients with PDAC.

We also observed a broad expression of Shh-pathway molecules in our patients, which emphasized the important role of Shh signaling in pancreatic cancer 7. We showed that a strongly activated Shh pathway with high expression levels of SMO and Gli1 was independent prognostic factors for PDAC, consistent with recently reported results 31, 32. However, we found that expression levels of Gli2 and Shh did not correlate with survival outcomes, in contrast to previous studies 31, 32. This discrepancy may be due to our study's limited sample size and data heterogeneity.

Studies have suggested that specific driver gene mutations reprogram the tumor microenvironment via the Shh pathway to form a dense stroma in pancreatic cancer. The oncogenic *KRAS* 16 and mutational inactivation of *TP53* 17 activate Shh signaling. However, the relationship between the Shh pathway and SMAD4 mutation has rarely been reported. SMAD4 mutations are relatively specific in pancreatic cancer and are central mediators of the transforming growth factor beta (TGF-β) signaling pathway 34. The TGF-β/smad4 pathway plays a tumor suppressive effect in normal pancreatic cells, which plays an important role in the development of tumors 34. In SMAD4-deficient pancreatic cancer, the accumulation of TGF-β leads to the release of extracellular molecule, such as MMP2, MMP9 35. Overexpression of TGF-β is also a major factor in fibrosis in many tumors 36, as the Shh pathway is widely recognized as the classic pathway for the formation of extracellular matrix in pancreatic cancer 29. Therefore, there may be a connection between the two pathways. During embryonic development, TGF-β family members are involved in the induction of pancreatic differentiation, meanwhile inhibiting the local expression of the transcription factor Shh 37. This suggests a relation between TGF-β/smad4 and Shh pathway early in the embryo. Thus, we hypothesized that the inhibitory effect of TGF-β/smad4 on Shh might be attenuated due to the *SMAD4* deficient in pancreatic cancer, and the higher Shh expression strongly activated the Shh pathway, which appears to promote tumor development. The hypothesis is consistent with previous reports that both TGF-β/smad4 signaling and Shh pathway can promote tumor growth through epithelial mesenchymal transition 38. In addition, it has also been confirmed in a special animal model that Shh ligands could induce TGF-β1 39. This association between Shh and TGF-β may also provide another possible hypothesis for the failure of studies targeting the Shh pathway, such as IPI-926, cyclopamine 40, 41. The treatment might cause the aberrant over-expression of Shh downstream molecule, and induce more TGF-β1, which possibly associated with an increased propensity of PDAC to metastasize. Similarly, the loss of SMAD4 was also confirmed to be associated with metastasis of pancreatic cancer 29. Interestingly, *SMAD4* inactivation in the pancreatic exocrine cells enhances fibrotic responses, possibly via upregulation of Shh RNA expression 33. We also showed that the mutational status of *SMAD4* was important for the activation of Shh signaling, which is a key pathway to promote desmoplasia 6, 7. The loss of Smad4 was significantly associated with high expression levels of Shh and Gli1. Patients with a combined status of activated-Shh-pathway^strong^ /*SMAD4*^lost^ showed the poorest survival outcomes compared with the other patients in our study. Our results, together with previous reports, suggested that specific driver gene mutations program the desmoplasia process. Further studies are needed to validate this hypothesis.

Some limitations of our study include smaller sample size and bias due to single-center and retrospective data. Future studies with larger patient numbers may validate the prognostic significance of the genetic status of driver genes and expression of Shh-pathway molecules in PDAC. Our study conclusions are only based on immunohistochemical data to provide insights into potential prognostic biomarkers for PDAC. Our statistical analysis does not fully differentiate between correlation and causation. Future studies are needed to comprehensively analyze the genetic and molecular basis of our observations to fully understand the underlying molecular mechanisms.

## Conclusions

We showed that the loss of *SMAD4* was associated with an activated Shh signaling pathway in resectable pancreatic cancer. This correlation may be a predictive factor to enable better prognosis in patients with PDAC and facilitate patient counseling and disease management.

## Supplementary Material

Supplementary figures and tables.

## Figures and Tables

**Table 1:** Univariate and Multivariate Cox regression for Survival analysis

Variables	RFS		OS	
	HR (95% CI)	*p* value	HR (95% CI)	*p* value
**Univariate analysis**				
** Smad4 (lost *vs.* intact)**	1.886 (1.391-2.558)	0.000*	1.887 (1.406-2.532)	0.000*
** TP53 (abnormal *vs.* normal)**	1.173 (0.881-1.562)	0.275	1.436 (1.086-1.901)	0.011*
** p16 (lost *vs.* intact)**	0.918 (0.667-1.263)	0.599	1.041 (0.770-1.408)	0.739
** Shh (high *vs.* low)**	0.766 (0.581-1.011)	0.059	0.864 (0.664-1.124)	0.227
** Gli1 (high *vs.* low)**	1.716 (1.301-2.263)	0.000*	1.988 (1.517-2.604)	0.000*
** Gli2 (high *vs.* low)**	0.975 (0.739-1.285)	0.885	1.018 (0.782-1.306)	0.895
** SMO (high *vs.* low)**	1.436 (1.078-1.911)	0.013*	1.411 (1.077-1.848)	0.013*
** Age (>60 *vs.* ~ 60 y)**	1.091 (0.830-1.434)	0.534	1.091 (0.837-1.421)	0.520
** Gender (male *vs.* female)**	1.706 (1.288-2.256)	0.000*	1.663 (1.265-2.185)	0.000*
** Serum CA19-9 (>37 *vs.* ~ 37 U/mL)**	2.476 (1.747-3.508)	0.000*	2.524 (1.828-3.486)	0.000*
** Location (body and tail* vs.* head)**	1.804 (1.356-2.400)	0.000*	1.671 (1.266-2.205)	0.000*
** Largest tumor size (>2 *vs.* ~ 2 cm)**	0.940 (0.646-2.369)	0.794	1.509 (1.038-2.195)	0.030*
** Lymph node metastasis (positive *vs.* negative)**	1.247 (0.951-1.635)	0.110	1.437 (1.106-1.867)	0.007*
** Venous invasion (positive *vs.* negative)**	1.739 (1.235-2.449)	0.002*	2.331 (1.654-3.284)	0.000*
** Perineural invasion (positive *vs.* negative)**	1.293 (0.887-1.885)	0.182	0.956 (0.681-1.342)	0.795
** Grading (poor *vs.* well/moderate)**	1.516 (1.219-2.037)	0.006*	1.573 (1.185-2.087)	0.002*
** UICC T (T3-4 *vs.* T1-2)**	1.629 (1.238-2.144)	0.000*	2.379 (1.811-3.127)	0.000*
** UICC N (N1-2 *vs.* N0)**	1.247 (0.951-1.635)	0.110	1.437 (1.106-1.867)	0.007*
** UICC stage**		0.001*		0.000*
**I**	1		1	
**II**	1.134 (0.826-1.556)	0.438	1.560 (1.154-2.108)	0.004
**III**	3.417 (2.340-4.990)	0.001	4.144 (2.845-6.037)	0.000
**Multivariate analysis**				
** Smad4 (lost *vs.* intact)**	NS	0.074	1.551 (1.009-2.384)	0.045*
** TP53 (abnormal *vs.* normal)**	NA		NS	0.149
** Gli1 (high *vs.* low)**	1.590 (1.187-2.131)	0.002*	1.541 (1.071-2.217)	0.020*
** SMO (high *vs.* low)**	1.776 (1.273-2.450)	0.001*	1.782 (1.283-2.474)	0.001*
** Gender (male *vs.* female)**	2.457 (1.800-3.355)	0.000*	2.208 (1.614-3.022)	0.000*
** Serum CA19-9 (>37 *vs.* ~ 37 U/mL)**	2.849 (1.963-4.135)	0.000*	3.190 (2.203-4.621)	0.000*
** Location (body and tail* vs.* head)**	2.086 (1.544-2.818)	0.000*	1.821 (1.331-2.492)	0.000*
** Largest tumor size (>2 *vs.* ~ 2 cm)**	NA		NS	0.359
** Lymph node metastasis (positive *vs.* negative)**	NA		1.638 (1.221-2.197)	0.001*
** Venous invasion (positive *vs.* negative)**	NS	0.710	2.020 (1.364-2.991)	0.000*
** Grading (poor *vs.* well/moderate)**	NS	0.937	NS	0.606

*p < 0.05; SMO: smoothened; Gli: glioma-associated oncogene homolog; OS: overall survival; RFS: recurrence-free survival; HR: hazard ratio; CI: confidence interval; NS: non-significant; NA: non-adoption; UICC: International Union against Cancer.

**Table 2:** Predictive model based on the combination of activated Shh Pathway and *SMAD4*

Variable	No.	RFS			OS		
	Patients	M-RFS (m)	HR (95% CI)	*p* value	M-OS (m)	HR (95% CI)	*p* value
**Group I**	36	12.0	1	0.000	34.0	1	0.000
**Group II**	141	6.7	2.005 (1.314-3.061)	0.001	12.8	2.019 (1.366-2.983)	0.000
**Group III**	60	5.1	2.976 (1.874-4.724)	0.000	9.0	3.598 (2.307-5.612)	0.000

OS: overall survival, RFS: recurrence-free survival, M-OS: median overall survival, M-RFS: median recurrence-free survival, HR: hazard ratio, CI: confidence interval

## Abbreviations

PDAC: pancreatic ductal adenocarcinoma
Shh: sonic hedgehog
SMO: smoothened
OS: overall survival
HR: hazard ratio
RFS: recurrence-free survival
Gli: glioma-associated oncogene
SMAD4/DPC4: decapentaplegic homolog 4
CDKN2A/P16: cyclin dependent kinase inhibitor 2A
UICC: Union for international cancer control
AJCC: American joint committee for cancer
IHC: immunohistochemistry
C-index: concordance index
AIC: Akaike information criterion
