# Supplementary material for: The Loss of SMAD4/DPC4 Expression Associated with a Strongly Activated Hedgehog Signaling Pathway Predicts Poor Prognosis in Resected Pancreatic Cancer
Source: J Cancer. 2019 Jul 10;10(17):4123–31. doi: 10.7150/jca.30883 (PMC6692626; doi:10.7150/jca.30883)

Supplementary tables and figures

Table S1. Patient and tumor characteristics

Characteristics	N (%). Patients
**Age, y [mean (SD)]**	61 (39-83)
**Gender**	
**Male**	132 (55.7)
**Female**	105 (44.3)
**Serum CA19-9**	
**~37 U/mL**	99 (41.8)
**>37 U/mL**	138 (58.2)
**Location**	
**Head**	99 (41.8)
**Body and tail**	126 (53.2)
**Other**	12 (5.0)
**Size, mm [mean (SD)]**	42 (1-97)
**Lymph node metastasis**	
**Negative**	129 (54.4)
**Positive**	108 (45.6)
**Venous invasion**	
**Negative**	195 (82.3)
**Positive**	42 (17.7)
**Perineural invasion**	
**Negative**	45 (19.0)
**Positive**	192 (81.0)
**Histologic grading**	
**Well -Moderate**	165 (69.6)
**Poor**	72 (30.4)
**UICC T**	
**T1**	33 (13.9)
**T2**	97 (40.9)
**T3**	89 (37.6)
**T4**	18 (8.0)
**UICC N**	
**N0**	129 (54.4)
**N1**	75 (31.6)
**N2**	33 (13.9)
**UICC stage**	
**I**	81 (34.2)
**II**	105 (44.3)
**III**	51 (21.5)

Data are expressed as percentages unless otherwise stated

SD: standard deviation.

Table S2. Immunohistochemistry of common driven genes and Shh protein expression

**Variables**	**N (%). Patients**
**Driver gene**	
**Smad4**	
**Lost**	168 (70.9)
**Intact**	69 (29.1)
**p53**	
**Normal**	90 (38.0)
**Abnormal**	147 (62.0)
**P16**	
**Lost**	177 (74.7)
**Intact**	60 (25.3)
**Kras**	237 (100.0)
**Sonic hedgehog pathway**	
**Shh**	
**Low**	135 (57.0)
**High**	102 (43.0)
**Gli1**	
**Low**	123 (51.9)
**High**	114 (48.1)
**Gli2**	
**Low**	102 (43.0)
**High**	135 (57.0)
**SMO**	
**Low**	90 (38.0)
**High**	147 (62.0)

SMO: smoothened; Gli: glioma-associated oncogene homolog.

Table S3. Relationship between Smad4, Gli1 and SMO protein expression and clinicopathologic characteristics using χ2 test

Characteristics	Smad4 immunoreactivity			Gli1 immunoreactivity			SMO immunoreactivity		
	Intact	Lost	*p* value	Low	High	*p* value	Low	High	*p* value
**Age, y**			0.421			0.100			0.250
**～60**	36	78		69	45		39	75	
**>60**	33	90		54	69		51	72	
**Gender**			0.015*			0.897			0.001*
**Male**	30	102		69	63		63	69	
**Female**	39	66		54	51		27	78	
**Serum CA19-9**			0.003*			0.490			0.329
**~37 U/ml**	39	60		54	45		34	65	
**>37 U/ml**	30	108		69	69		56	82	
**Location**			0.000*			0.006*			0.115
**Head**	42	57		63	36		30	69	
**Body and tail**	24	102		57	69		51	75	
**Other**	3	9	NA	3	9	NA	9	3	NA
**Largest Size**			0.028*			0.054			0.171
**~2 cm**	16	20		24	12		10	26	
**>2 cm**	53	148		99	102		80	121	
**Lymph node metastasis**			0.655			0.187			0.178
**Negative**	36	93		72	57		54	75	
**Positive**	33	75		51	57		36	72	
**Venous invasion**			0.227			0.196			0.739
**Negative**	60	135		105	90		75	120	
**Positive**	9	33		18	24		15	27	
**Perineural invasion**			0.489			0.227			0.476
**Negative**	15	30		27	18		15	30	
**Positive**	54	138		96	96		75	117	
**Histologic grading**			0.064			0.001*			0.100
**Well -Moderate**	54	111		99	66		57	108	
**Poor**	15	57		24	48		33	39	
**UICC T**			0.040*			0.026*			0.921
**T1+2**	45	85		76	54		49	81	
**T3+4**	24	83		47	60		41	66	
**UICC N**			0.614			0.252			0.001*
**N0**	36	93		72	57		54	75	
**N1**	21	54		33	42		36	39	
**N2**	12	21		18	15		0	33	
**UICC Stage**			0.569			0.162			0.003*
**I**	26	55		49	32		35	46	
**II**	31	74		50	55		46	59	
**III**	12	39		24	27		9	42	

*, p<0.05; UICC: International Union against Cancer. SMO: smoothened; Gli: glioma-associated oncogene homolog; NA: non-adoption.

Table S4. Relation between Smsd4 and Sonic Hedgehog Pathway Using $\chi2$ Test

**Variable**	**Smad4 Immunoreactivity**			
	Intact（n）	Loss（n）	*p* value	Pearson's R
**Shh**			0.000	0.343
**Low**	21	114		
**High**	48	54		
**Gli1**			0.000	0.505
**Low**	63	60		
**High**	6	108		
**Gli2**			0.340	
**Low**	33	69		
**High**	36	99		
**SMO**			0.516	
**Low**	24	66		
**High**	45	102		

SMO: smoothened; Gli: glioma-associated oncogene homolog.

Table S5. Multivariate Cox Regression for Survival Analysis Using the Combination of The Status of *SMAD4* and the SHH Pathway

**Variables**		**RFS**		**OS**	
		HR (95% CI)	*p* value	HR (95% CI)	*p* value
**Gender**	**Famale**	1	0.000*	1	0.000*
	**Male**	2.436 (1.793–3.309)		2.296 (1.689–3.122)	
**Serum CA19–9**	**~37 U/ml**	1	0.000*	1	0.000*
	**>37 U/ml**	2.854 (1.967–4.141)		3.312 (2.318–4.732)	
**Location**	**Head**	1	0.000*	1	0.000*
	**Body and Tail**	2.101 (1.551–2.846)		2.044 (1.516–2.756)	
**Largest tumor size**	**~ 2 cm**	NA		NS	0.327
	**> 2 cm**				
**Lymph node metastasis**	**Negative**	NA		1	0.005*
	**Positive**			1.521(1.138–2.032)	
**Venous invasion**	**Negative**	NS	0.692	1	0.002*
	**Positive**			1.869 (1.218–2.851)	
**Histologic grading**	**Well -Moderate**	NS	0.985	NS	0.800
	**Poor**				
TP53	**Normal**	NA		NS	0.088
	**Abnormal**				
**Group**	**I**	1	0.000*	1	0.000*
	**II**	1.813 (1.172–2.806)	0.008*	1.864 (1.218–2.851)	0.004*
	**III**	2.853 (1.755–4.639)	0.000*	3.309 (2.035–5.382)	0.000*

OS: overall survival; RFS: recurrence-free survival; HR: hazard ratio; CI: confidence interval; NS: non-significant; NA: non-adoption.

Table S6. Comparison of the Prognositic Accuracies of *SMAD4*, Activated SHH Pathway and the Combined Model

**Model**	**OS**				**RFS**			
	C-Index	AIC	BIC	*p* value	C-Index	AIC	BIC	*p* value
SMAD4	0.5862	2052	2056	0.000	0.5830	1927	1930	0.000
**Activated SHH Pathway**	0.5684	2045	2049	0.000	0.5548	1922	1926	0.000
***SMAD4*+activated SHH Pathway**	0.6220	2033	2037	0.000	0.6076	1914	1918	0.000

Table S7. Antibodies

Antibodies	Company	Dilution
**Rabbit anti-Kras polyclonal antibody ab180772**	Abcam	1:100
**Rabbit anti-p16 monoclonal antibody ab108349**	Abcam	1:50
**Rabbit anti-p53 polyclonal antibody ab31333**	Abcam	1:50
**Mouse anti-Smad4 monoclonal antibody sc-7966 B**	Santa Cruz Biotechnology	1:200
**Rabbit anti-Shh polyclonal antibody sc-9024**	Santa Cruz Biotechnology	1:100
**Rabbit anti-Gli1 polyclonal antibody #43926**	SAB	1:100
**Rabbit anti-SMO polyclonal antibody 20787-1-AP**	Proteintech	1:100
**Mouse anti-Gli2 monoclonal antibody sc-271786**	Santa Cruz Biotechnology	1:200

Table S8. Relation between other driver genes and Sonic Hedgehog Pathway Using χ2 Test

	TP53			***P16***		
	Abnormal	Normal	*p* value	Lost	Intact	*p* value
**Shh**			0.090			0.079
**Low**	90	45		95	40	
**High**	57	45		82	20	
**Gli1**			0.647			0.797
**Low**	78	45		91	32	
**High**	69	45		86	28	
**Gli2**			0.018			0.582
**Low**	72	30		78	24	
**High**	75	60		99	36	
**SMO**			0.615			0.194
**Low**	54	36		63	27	
**High**	93	54		114	33	

SMO: smoothened; Gli: glioma-associated oncogene homolog.

Fig. S1 Immunohistochemical Labeling of Driver Genes

A1) Smad4 were scored as intact (positive), indicating the presence of an intact gene, **A2)** Smad4 were scored as lost (negative), indicating a deletion or inactivating mutation of the gene had occurred. **bB** Immunohistochemical labeling of Kras was all scored as intact (positive), indicating the presence of an intact gene. **C1)** Pancreatic ductal adenocarcinoma (PDAC) showing a “normal” pattern of p53 immunohistochemical labeling. **C2)** Example of PDAC with diffusely positive nuclear labeling for p53, namely robust nuclear accumulation of immunolabeled protein in ≥30% of neoplastic cells compared with adjacent normal cells. **C3)** Example of PDAC with loss of nuclear labeling for p53, namely a virtual absence of immunolabeling compared with adjacent normal tissue (immunolabeling in <5% of neoplastic cells), suggesting the presence of an intragenic deletion, nonsense or frameshift mutation. **D1)** Positive p16 im- munolabeling indicating an intact CDKN2A/p16 gene. **D2)** Negative p16 immunolabeling indicating alteration of the CDKN2A/p16 gene.

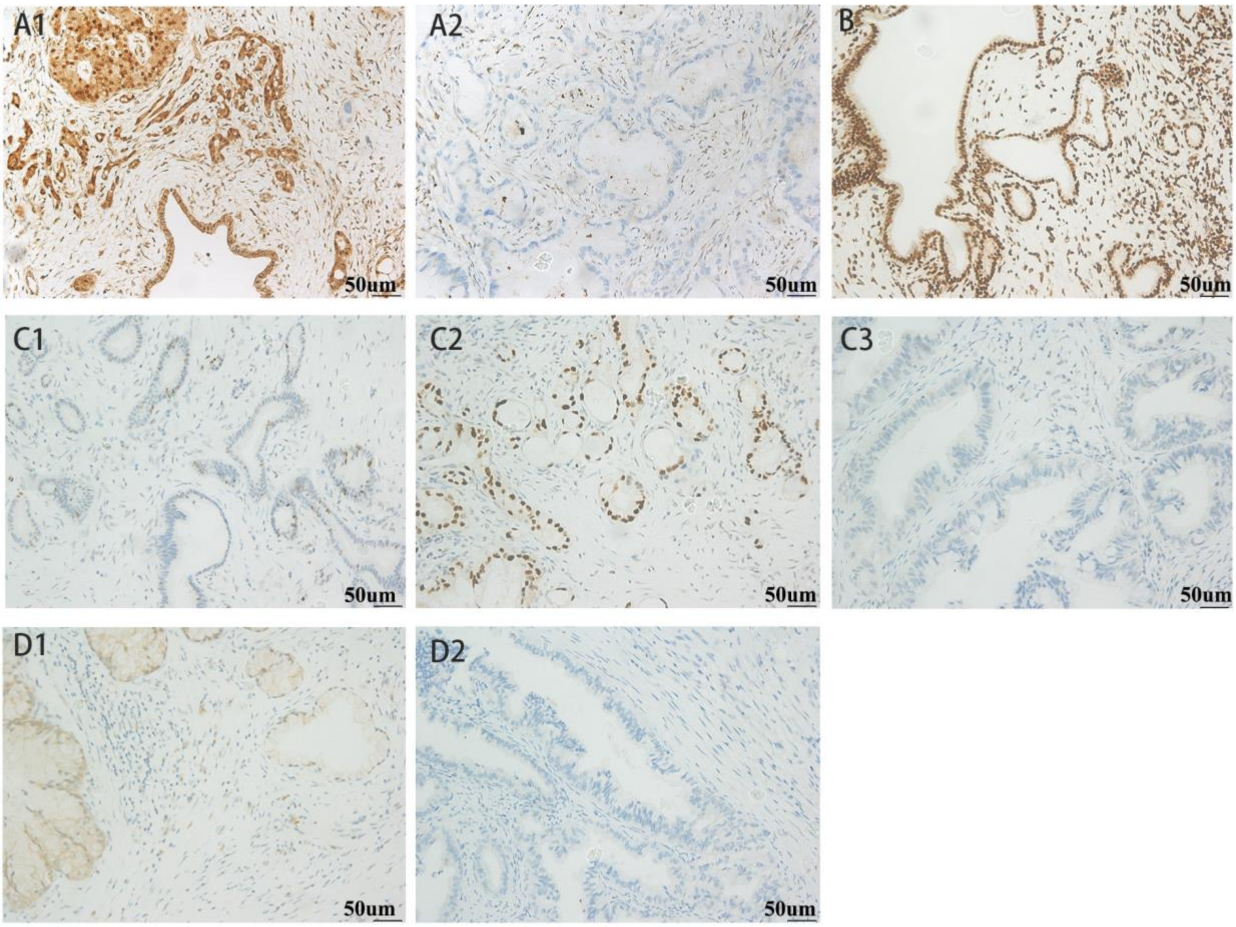

Fig. S2 Immunohistochemical Labeling of Shh and Gli2 Expression in Pancreatic Cancer

Shh: **A1)** weak expression, **A2)** moderate expression, and **A3)** intense expression in tumor cells. Gli2: **B1)** weak expression, **B2)** moderate expression, and **B3)** intense expression in tumor cells and stroma. All magnification = 400×. Positive staining appears brown.

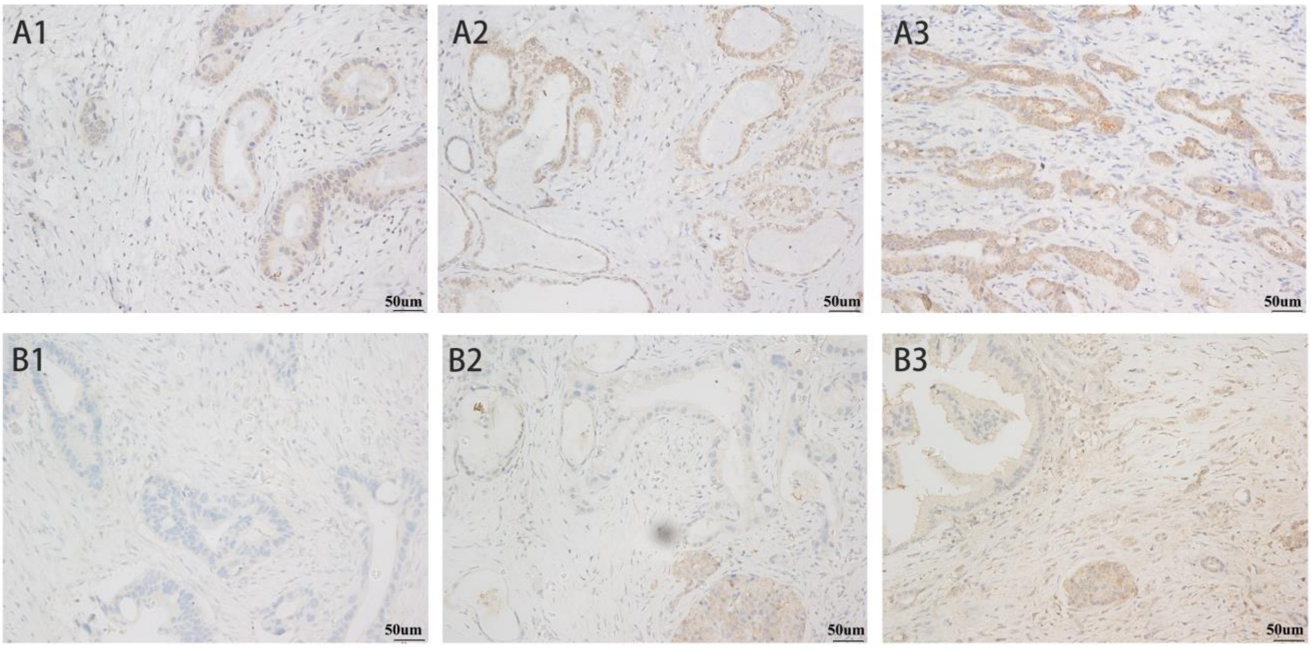

Supplement: Supplementary file 1 — Supplementary figures and tables. [file jcav10p4123s1.pdf]
